# Physical activity and recreational screen time among Chinese children and adolescents: a national cross-sectional study

**DOI:** 10.3389/fpubh.2024.1376330

**Published:** 2024-07-10

**Authors:** Mingming Guo, Yangming Zhu, Xiaozan Wang

**Affiliations:** College of Physical Education and Health, East China Normal University, Shanghai, China

**Keywords:** children and adolescents, physical activity, recreational screen time, Chinese, cross-sectional study

## Abstract

**Background:**

The increasing prevalence of physical inactivity and prolonged Recreational Screen Time (RST) among children and adolescents is emerging as a significant public health concern. This study investigates the current status of Physical Activity (PA) and RST among Chinese children and adolescents from 2017 to 2019. It also examines variations in PA and RST across different school levels, genders, urban–rural areas, regions, and seasons.

**Methods:**

A national cross-sectional survey, conducted in China from 2017 to 2019, included 53,101 children and adolescents from grades 4 to 12 (aged 10 to 18 years old). Data on PA and RST were collected via self-administered questionnaires. The study employed descriptive statistics, calculated weighted prevalence rates, and conducted differential analysis across diverse demographic groups.

**Results:**

Between 2017 and 2019 in China, merely 28.73% of children and adolescents adhered to World Health Organization’s PA guidelines, while 76.09% met China’s RST guidelines. Notably, females, higher-grade students, rural residents, and children and adolescents from southern regions exhibited significantly lower levels of PA compared to their male, lower-grade, urban, and northern counterparts. Concurrently, RST was significantly higher among males, lower-grade students, rural residents, and those from northern regions. Seasonal variations were also observed, with lower PA and higher RST in autumn and winter as compared to spring.

**Conclusion:**

The study reveals a concerning low level of PA among Chinese children and adolescents, with marked disparities in PA and RST across different groups. This underscores the need for targeted health promotion strategies to enhance PA and mitigate RST among various child and adolescent populations.

## 1 Introduction

Physical activity (PA), defined as any bodily movement that results in energy expenditure in skeletal muscles, such as walking, cycling, exercising, or doing household chores (1). Recreational Screen Time (RST), on the other hand, encompasses discretionary screen activities conducted while sedentary, such as watching television, playing video games, and using computers (2). Both PA and RST significantly influence the health and well-being of children and adolescents. Extensive research indicates that higher levels of PA, coupled with reduced RST, are effective in preventing and managing various health issues, including obesity and type 2 diabetes (3). Additionally, adequate PA and less RST enhances physical health, boosts cognitive abilities, improves academic performance, and reduces mental stress (3).

The World Health Organization (WHO) recommends that children and adolescents should engage in at least 60 min of Moderate-to-Vigorous Physical Activity (MVPA) daily to be considered active (3). Similarly, health guidelines from countries like Canada and China advocate for limiting RST to under 2 h per day for this demographic (2, 4). Despite these recommendations, adherence rates among children and adolescents are alarmingly low globally – only about 27 to 33% meet the WHO’s PA guidelines, and a mere 34 to 39% adhere to the RST guidelines of Canada and China (5, 6).

The same concerning phenomenon of low levels of PA and increased RST is also observed amongst Chinese children and adolescents, particularly in the context of rapid social transformation, economic growth, and widespread adoption of personal electronic devices in China (7, 8). Notably, in the Global Report Card on Physical Activity for Children and Youth, China ranks among the lowest in terms of PA and RST indicators (9). To support the Chinese government and organizations in formulating policies and initiatives to promote PA and reduce RST in children and adolescents, several nationally representative studies have examined the prevalence and current state of PA and RST among this demographic. For instance, Fan et al.’s (10) survey revealed that approximately 30% of Chinese children and adolescents met the WHO’s PA guidelines. Similarly, Zhu et al.’s (11) study found that 34.1% of Chinese children and adolescents adhered to the WHO’s PA guidelines, and 65.4% adhered to China’s RST guidelines, respectively. However, Liu et al.’s (12) survey indicated a significant decline, with only 14% of participants meeting the WHO’s PA guidelines and just 51% adhering to China’s RST guidelines. These studies also indicated variations in PA and RST based on sociodemographic factors such as gender and age. The sharp decrease in adherence between 2017 and 2020 might be partly attributed to the pandemic, but the lack of comprehensive data from 2017 to 2020 hinders a thorough understanding of the current state and hampers the development of effective policies.

To bridge this gap, our study, under the auspices of the Construction of a Big Data Platform for Children and Adolescents’ Sports and Fitness in China (CBDPCASF) project, assessed the current status of PA and RST among Chinese children and adolescents during 2017–2019. The objectives were twofold: (1) to augment existing data regarding PA and RST among this population during the specified period, and (2) to elucidate the distribution of PA and RST across various demographics, including gender, school levels, urban–rural areas, regions, and seasonal variations.

## 2 Materials and methods

### 2.1 Study design and participants

This study was a component of the CBDPCASF project. Funded by the National Social Science Foundation of China, the CBDPCASF is a major national initiative aimed at gathering data on the sports participation and health of Chinese children and adolescents. Between 2017 and 2019, the project conducted three cross-sectional surveys. Each annual survey utilized stratified sampling methods, reflecting the distribution of student populations across various school levels in China. Schools participating in the CBDPCASF project, encompassing primary, middle, and high schools, were selected for the study. Subsequently, children and adolescents in grades 4 to 12 (aged 10 to 18 years old) from these schools were invited to participate. The study received approval from the Human Experimentation Ethics Committee at East China Normal University (Registration Code: HR 222–2019) and was conducted in strict accordance with the principles outlined in the Declaration of Helsinki. Furthermore, the research adhered to the STROBE guidelines for cross-sectional studies.

Over these 3 years, a total of 53,101 students from 150 schools in 18 provinces participated. This included 27,555 students from 120 primary schools, 18,115 students from 63 middle schools, and 7,431 students from 28 high schools.

### 2.2 Procedures

In September of each year from 2017 to 2019, the CBDPCASF project team randomly selects a variety of primary, middle, and high schools from those participating in the CBDPCASF project, according to the proportion of students at different school levels in China. These schools are invited to participate in this study, and are free to choose when to conduct the survey anytime from September of the current year to June of the following year. During the selected time periods, invitations for the survey are extended to students in grades 4 to 12 through their schools. Teachers inform the students about the purpose of the study as well as its potential risks and benefits, and informed consent is also obtained from willing students and their guardians.

After consent was secured, trained research assistants distributed online questionnaires to the students using the Questionnaire Star platform (13). The questionnaires were completed by the students via smartphones or computers. Post data collection, the research assistants classified the area status (urban or rural) and regional location (north or south) of each student’s school using Baidu Maps and the Qinling-Huaihe Line. Baidu Maps, the largest map service provider in China, can determine whether a location is urban or rural based on the name of the place entered (14). The Qinling-Huaihe Line is a geographical divide in China, with regions north of this line typically having drier, colder climates, poorer air quality, and lower levels of economic development compared to regions south of the line (15). The season of each student’s survey participation was determined based on the completion date of the questionnaire, with March–May classified as spring, June–August as summer, September–November as autumn, and December–February as winter. After collecting data over 3 years, data from annual surveys conducted between 2017 and 2019 were consolidated to form the dataset for this study.

### 2.3 Instruments

To assess participants’ PA, the study employed the Chinese adaptation of the Physical Activity Questionnaire for Older Children (PAQ-C) (16). The PAQ-C, a self-report instrument, evaluates PA over a 7-day recall period. Its reliability and validity in assessing PA among Chinese children and adolescents are well-established (17, 18). The questionnaire comprises 10 items, beginning with questions about the frequency of engagement in various PA such as swimming, martial arts, and basketball. Items 2 to 8 inquire about PA during specific periods, including physical education classes, recess, after-school hours, evenings, and weekends. The ninth question probes the frequency of engaging in more than 30 min of PA daily over the past week, while the final item addresses any special circumstances that may have limited PA in the preceding week. The first 9 items utilize a five-point Likert scale, ranging from 1 (lowest level or frequency of PA) to 5 (highest level or frequency of PA). The last question presents a binary choice, “Yes” or “No,” to indicate whether any special circumstances last week restricted the participants’ engagement in PA. The final PA score was s calculated as the average of scores from questions 1 to 9, reflecting the participants’ level of PA over the past 7 days (16). Furthermore, according to a comparative study of PAQ-C and accelerometer data by Voss et al. (17), a PAQ-C score exceeding 2.87 corresponds to a daily MVPA duration of over 60 min. Consequently, this study classified a PAQ-C score above 2.87 as compliant with the WHO’s PA guidelines.

Participants’ RST was evaluated using the Chinese adaptation of the Adolescent Sedentary Activity Questionnaire (ASAQ) (19). The ASAQ, a 7-day recall tool, has proven reliable and valid for studying sedentary time among Chinese children and adolescents (19, 20). It consists of 6 items that assess RST on weekdays and weekends, focusing on time spent watching TV, movies, and using smartphones, computers, or tablets (excluding educational or work purposes). The calculation of participants’ weekly RST is achieved by summing the total of the 6 items, and the average daily RST is obtained by dividing the weekly RST by 7 (19). According to the RST guidelines of Canada and China (2, 4), participants with less than 2 h of daily RST are considered to have met the RST guidelines.

Demographic information, including school, gender, and school level, was also collected during the survey.

### 2.4 Statistical analyses

Participants with incorrect (3,809), outlier (608, based on Tukey’s method) (21), or irrelevant responses (those unable to engage in PA due to special circumstances or pre-existing medical conditions, totaling 3,151) were excluded from the analysis.

The representation factor for each student was determined using the 2019 data on total students across different school levels, as provided by the Ministry of Education of China (22). This factor was then applied as a weighting variable in subsequent analyses. Weighted percentages for the overall sample were calculated based on gender, area, region, and season. Unweighted percentages for different school level samples were also calculated based on gender, area, region, and season. Both unweighted and weighted prevalence rates for adherence to PA and RST guidelines, along with their 95% confidence intervals (CIs), were calculated across gender, area, region, and season. A Generalized Linear Model, both weighted and unweighted and adjusted for demographic covariates (gender, area, region, season, and school level), was employed to analyze the difference in adherence to PA and RST guidelines.

All analyses were conducted using Python (Python 3.10, Python Software Foundation, Wilmington, DE, United States) with the statsmodels, pandas, and scipy libraries (23). A two-sided significance level was set at 0.05.

## 3 Results

### 3.1 Sample characteristics

Following the data cleaning process, the final analysis included a total of 45,533 participants, consisting of 24,040 primary school students, 15,081 middle school students, and 6,412 high school students. Of these, the weighted proportion of female participants was 48.66%, rural participants accounted for 18.93%, and participants from the southern region comprised 39.48%. The proportions of participants surveyed in spring, autumn, and winter were 46.43, 24.96, and 28.61%, respectively. The unweighted sample sizes divided by gender, area, region, season, and school level, along with their corresponding weighted or unweighted percentages, are detailed in Table 1.

**Table 1:** Unweighted sample size, with corresponding weighted and unweighted percentages of school level participation.

	No. of participants by school level (Weight %)^a^			
	All school level	Primary school	Middle school	High school
Overall	45,533	24,040 (100%)	15,081 (100%)	6,412 (100%)
Gender				
Male	23,321(51.34%)	12,631 (52.54%)	2,993 (46.68%)	7,697 (51.04%)
Female	22,212(48.66%)	11,409 (47.46%)	3,419 (53.32%)	7,384 (48.96%)
Area^b^				
Urban	36,831 (81.07%)	19,705 (81.97%)	5,219 (81.39%)	11,907 (78.95%)
Rural	8,702 (18.93%)	4,335 (18.03%)	1,193 (18.61%)	3,174 (21.05%)
Region^c^				
North	17,042 (60.52%)	12,353 (51.39%)	3,232 (50.41%)	12,906 (85.58%)
South	28,491 (39.48%)	11,687 (48.61%)	3,180 (49.59%)	2,175 (14.42%)
Season^d^				
Spring	13,032 (46.43%)	10,742 (44.68%)	3,570 (55.68%)	6,882 (45.63%)
Autumn	21,194 (24.96%)	6,689 (27.82%)	478 (7.45%)	4,140 (27.45%)
Winter	11,307 (28.61%)	6,609 (27.49%)	2,364 (36.87%)	4,059 (26.91%)

a The all school level percentages were calculated based on the proportion of students in different school levels in 2019, with the percentages for different school levels not being weighted.

b Area classifications are based on the urban or rural status of each school, as designated by the Baidu Maps Open Platform.

c Regions were delineated by the natural geographic boundaries of China, specifically the Qinling-Huaihe Line.

d The season was determined based on different months: March–May for spring, June–August for summer, September–November for autumn, and December–February for winter. Since summer is the school holiday period for students, no survey was conducted during this season.

### 3.2 Prevalence and differences in meeting PA guidelines

Between 2017 and 2019, only 28.57% of children and adolescents met the PA guidelines. A trend of decreasing adherence was observed with increasing school levels: 32.85% in primary school, 23.65% in middle school, and only 19.67% in high school. The detailed weighted and unweighted prevalence rates for different genders, areas, regions, and seasons are presented in Table 2.

**Table 2:** Weighted and unweighted prevalence of meeting PA and RST guidelines among chinese children and adolescents by gender, area, region, season, and school level.^a^

	All school level	Primary school	Middle school	High school
Meeting PA guidelines (95% CI)^b^				
Overall	28.57 (28.15–28.98)	32.85 (32.26–33.44)	23.65 (22.97–24.32)	19.67 (18.69–20.64)
Gender				
Male	33.90 (33.29–34.52)	37.34 (36.50–38.19)	28.89 (27.88–29.91)	28.87 (27.24–30.49)
Female	23.04 (22.48–23.60)	27.87 (27.05–28.70)	18.17 (17.29–19.05)	11.61 (10.54–12.69)
Area				
Urban	29.39 (28.93–29.86)	34.03 (33.37–34.69)	23.88 (23.11–24.64)	20.16 (19.07–21.25)
Rural	24.86 (23.94–25.79)	27.50 (26.17–28.83)	22.78 (21.32–24.24)	17.52 (15.36–19.68)
Region				
North	28.47 (27.91–29.03)	32.50 (31.68–33.33)	23.57 (22.84–24.30)	20.61 (19.21–22.00)
South	28.78 (28.05–29.50)	33.22 (32.36–34.07)	24.09 (22.29–25.89)	18.71 (17.36–20.07)
Season				
Spring	29.21 (28.41–30.00)	34.76 (33.61–35.90)	22.76 (21.47–24.05)	17.81 (16.27–19.35)
Autumn	25.11 (24.25–25.97)	28.94 (27.86–30.03)	20.48 (19.25–21.71)	17.57 (14.16–20.99)
Winter	30.17 (29.55–30.80)	34.11 (33.21–35.01)	26.07 (25.03–27.11)	21.18 (19.84–22.52)
Meeting RST guidelines (95% CI)^b^				
Overall	81.61 (81.25–81.97)	80.58 (80.08–81.08)	81.33 (80.71–81.96)	86.67 (85.83–87.50)
Gender				
Male	79.46 (78.94–79.99)	78.63 (77.92–79.35)	78.67 (77.75–79.58)	84.70 (83.41–85.99)
Female	83.88 (83.39–84.37)	82.74 (82.05–83.44)	84.11 (83.28–84.95)	88.39 (87.31–89.46)
Area				
Urban	81.81 (81.41–82.21)	80.71 (80.16–81.26)	81.99 (81.30–82.68)	86.24 (85.31–87.18)
Rural	80.85 (80.00–81.69)	80.00 (78.81–81.19)	78.86 (77.44–80.28)	88.52 (86.71–90.33)
Region				
North	80.93 (80.44–81.42)	79.51 (78.80–80.22)	81.59 (80.92–82.26)	85.83 (84.63–87.03)
South	81.99 (81.35–82.62)	81.71 (81.01–82.42)	79.82 (78.13–81.50)	87.52 (86.37–88.66)
Season				
Spring	79.43 (78.73–80.14)	78.47 (77.48–79.46)	78.94 (77.68–80.19)	84.64 (83.19–86.10)
Autumn	82.56 (81.80–83.32)	82.75 (81.84–83.65)	80.56 (79.35–81.76)	85.77 (82.64–88.91)
Winter	82.29 (81.77–82.82)	80.53 (79.79–81.28)	83.22 (82.33–84.10)	88.12 (87.06–89.18)

a The all school level prevalence is weighed based on the proportion of students in different school levels in 2019, with the prevalence for different school levels not being weighted.

b CI, confidence interval.

In terms of associations as measured by odds ratios (ORs), female children and adolescents were significantly less likely to meet PA guidelines compared to males, with an OR of 0.570 (95% Confidence Interval [CI]: 0.569–0.571). Similarly, rural children and adolescents were less likely to adhere to PA guidelines than their urban counterparts, evidenced by an OR of 0.782 (95% CI: 0.780–0.783). Additionally, children and adolescents from southern regions demonstrated lower adherence compared to those from northern regions, with an OR of 0.986 (95% CI: 0.985–0.987). Seasonal variations were also evident; children and adolescents surveyed in winter and autumn were less likely to meet PA guidelines compared to those surveyed in spring, with ORs of 0.916 for winter (95% CI: 0.914–0.917) and 0.745 for autumn (95% CI: 0.744–0.746). Detailed associations between meeting PA guidelines and factors such as gender, area, region, and season, as measured by ORs, are presented in Table 3.

**Table 3:** Odds ratios for meeting PA and RST guidelines by gender, area, region, season, and school level.

	All school level	Primary school	Middle school	High school
Meeting PA guidelines (95% CI)				
Gender				
Male	1 [Reference]	1 [Reference]	1 [Reference]	1 [Reference]
Female	0.570 (0.569–0.571)	0.646 (0.612–0.683)	0.548 (0.507–0.592)	0.324 (0.284–0.369)
Area				
Urban	1 [Reference]	1 [Reference]	1 [Reference]	1 [Reference]
Rural	0.782 (0.780–0.783)	0.719 (0.666–0.775)	0.892 (0.809–0.983)	0.910 (0.761–1.089)
Region				
North	1 [Reference]	1 [Reference]	1 [Reference]	1 [Reference]
South	0.986 (0.985–0.987)	0.960 (0.906–1.017)	1.041 (0.935–1.160)	0.565 (0.467–0.683)
Season				
Spring	1 [Reference]	1 [Reference]	1 [Reference]	1 [Reference]
Autumn	0.745 (0.744–0.746)	0.748 (0.699–0.801)	0.748 (0.681–0.821)	0.434 (0.322–0.584)
Winter	0.916 (0.914–0.917)	1.012 (0.944–1.084)	0.816 (0.742–0.898)	0.472 (0.387–0.577)
Meeting RST guidelines (95% CI)				
Gender				
Male	1 [Reference]	1 [Reference]	1 [Reference]	1 [Reference]
Female	1.361 (1.360–1.363)	1.312 (1.230–1.400)	1.449 (1.333–1.574)	1.383 (1.197–1.599)
Area				
Urban	1 [Reference]	1 [Reference]	1 [Reference]	1 [Reference]
Rural	0.897 (0.895–0.898)	0.978 (0.898–1.066)	0.738 (0.666–0.818)	1.217 (0.985–1.503)
Region				
North	1 [Reference]	1 [Reference]	1 [Reference]	1 [Reference]
South	1.096 (1.094–1.097)	1.143 (1.068–1.224)	0.906 (0.808–1.017)	0.786 (0.617–1.000)
Season				
Spring	1 [Reference]	1 [Reference]	1 [Reference]	1 [Reference]
Autumn	0.979 (0.977–0.981)	1.136 (1.048–1.232)	0.811 (0.733–0.896)	0.668 (0.471–0.948)
Winter	0.824 (0.822–0.825)	0.900 (0.830–0.975)	0.689 (0.621–0.764)	0.606 (0.471–0.779)

### 3.3 Prevalence and differences in meeting RST guidelines

The results from 2017 to 2019 indicate that 81.61% of children and adolescents adhered to the RST guidelines. Notably, adherence to the guidelines increased with ascending school levels: 80.58% in primary school, 81.33% in middle school, and 86.67% in high school. Detailed weighted and unweighted prevalence rates for different genders, areas, regions, and seasons are available in Table 2.

In terms of associations as measured by ORs, the study found that female children and adolescents were more likely to meet the RST guidelines compared to males, with an OR of 1.361 (95% CI: 1.360–1.363). Rural children and adolescents were less likely to adhere to RST guidelines than their urban counterparts, as evidenced by an OR of 0.897 (95% CI: 0.895–0.898). Additionally, children and adolescents from southern regions showed higher adherence compared to those from northern regions, with an OR of 1.096 (95% CI: 1.094–1.097). Seasonally, adherence rates were lower in winter and autumn than in spring, with ORs of 0.824 for winter (95% CI: 0.822–0.825) and 0.979 for autumn (95% CI: 0.977–0.981). The associations between meeting RST guidelines and factors such as gender, area, region, and season, as measured by ORs, are detailed in Table 3.

## 4 Discussion

This study examined the PA and RST among Chinese children and adolescents, revealing that only 28.73% met the WHO’s PA guidelines, while 76.09% adhered to the RST guidelines of China and Canada. Notably, differences in PA and RST were observed across various demographics, including gender, area, region, and season, with these variances being more pronounced across some school levels.

Globally, previous research indicates that 27 to 33% of children and adolescents meet PA guidelines, but only 34 to 39% adhere to RST guidelines (6). The PA levels among Chinese children and adolescents align with the global average, however, their RST is significantly lower. In fact, the RST of Chinese children and adolescents appears to be indeed less than that of other countries. For instance, a 2017 national survey also reported that 65.4% of Chinese children and adolescents had less than 2 h of daily RST (11). This could be attributed to China’s status as a developing country with generally lower household incomes, leading to fewer children owning personal electronic devices. Additionally, the rigorous Chinese education system, particularly the emphasis on entrance exams for high schools and colleges, may compel students to dedicate more time to studies than screen-based recreation. Therefore, in China, greater efforts should be directed towards enhancing PA among children and adolescents.

Consistent with global trends, our study found that in China, males and younger children are more likely to meet PA guidelines, whereas females and older children are more likely to meet RST guidelines (5, 11, 24). The underlying reasons, explored in prior research, include physiological differences, interest disparities, varying levels of social support, shifts in academic pressure, environmental factors, and perceptions of PA (25–27). Despite these gender and age disparities, the harms of insufficient PA and excessive RST do not differ by gender or age, underscoring the need for targeted interventions focusing on the PA of females and older children, and the RST of males and younger students.

Studies by Cai et al. (28) and Song et al. (29) as well as Wagner et al. (30) demonstrate that urban children and adolescents have higher levels of PA and more RST compared to their rural counterparts, attributed to better access to PA facilities and electronic devices. Conversely, research by Euler et al. (31) and Sandercock et al. (32) shows that rural children and adolescents engage in higher levels of PA but less RST than those in urban areas. This difference is primarily due to rural children and adolescents having more space for PA, frequently needing to assist with family labor tasks, and having fewer electronic devices (31, 32). Distinct from both scenarios, our study indicates that compared to urban children and adolescents, those in rural areas exhibit both lower levels of PA and more RST. We believe that the higher levels of PA observed among urban children and adolescents in China, compared to their rural counterparts, corroborate the assumptions posited by Cai et al. (28) and Song et al. (29) Specifically, urban youths benefit from greater accessibility to PA facilities and have more opportunities for organized PA. However, the notably higher amounts of RST among rural children and adolescents compared to their urban counterparts may primarily be attributable to the phenomenon of parents from rural China working away from their hometowns (33), resulting in less supervision and potentially more time spent on electronic devices by these children. Therefore, prioritizing the enhancement of PA for rural children and adolescents through increased opportunities for PA, and managing their RST through heightened supervision, should be emphasized in China.

In China, the Qinling-Huaihe Line serves as a crucial geographical and economic boundary. Regions north of this line typically experience more arid and colder climates, poorer air quality, and lower levels of economic development compared to the south (15, 34). This study found that children and adolescents in the southern regions are less likely to meet PA guidelines but more likely to adhere to RST guidelines than those in the north. Guthold et al.’s (5) research indicates that children in economically affluent regions engage in more PA due to greater opportunities for such activities. Contrary to these findings, our study reveals that children in the economically advanced south demonstrate lower levels of PA. We hypothesize that this discrepancy may be attributable to the higher precipitation in southern China (15), which indirectly reduces the PA of children and adolescents (35). The relationship between RST, economic status, and regional disparities is more complex. Studies have produced varied findings, for instance, research by Demetriou et al. (36) suggests that in regions with higher levels of economic development, children and adolescents tend to have increased screen time (9). Conversely, Brodersen et al. (37) present findings that indicate more screen time among children and adolescents in regions with lower economic development. These differences could be related to the country where the study was conducted. In China, due to the pressures of entrance exams for high schools and colleges, affluent families typically enroll their children in various after-school tutoring classes (38). This leads to a reduction in their children’s RST, which may explain why the duration of RST among children and adolescents in southern China is less than in the northern regions. Therefore, in assessing the status of PA and RST among children and adolescents in different regions and formulating intervention measures, it is crucial to take into account the local economic, environmental, and social conditions.

Global studies indicate seasonal variations in PA and RST, with lower PA and higher RST observed in winter and autumn compared to spring, primarily due to more favorable springtime weather for PA (39–41). This study’s results confirm this, with children and adolescents surveyed in spring being more likely to meet both PA and RST guidelines compared to those surveyed in winter and autumn. This suggests that when weather conditions are less favorable, children’s and adolescents’ PA and RST should receive more attention, and conditions should be created to minimize the impact of climate on these behaviors, such as building more indoor activity spaces.

To our knowledge, this is the first study reporting on PA and RST among Chinese children and adolescents for the period 2017–2019, right before the pandemic. The study’s strengths include its nationally representative sample and consistent survey tools across different years. Most importantly, it provides an essential update on the status of PA and RST among Chinese children and adolescents during 2017–2019.

Despite these contributions, this study has several limitations. First, the self-reported nature of the survey tools might not accurately capture the actual PA and RST of children and adolescents, potentially leading to deviations in the calculation of prevalence rates. Although these inaccuracies do not significantly alter our understanding of the distribution of PA and RST, future studies could employ more objective measures, such as triaxial accelerometers, to enhance accuracy. Second, the PA questionnaire used in our study lacks detailed information regarding the duration and frequency of activities. As a result, our calculations of PA prevalence were based on criteria from Voss’s research, which limits their comparability with other studies. Future research should incorporate measures of intensity, frequency, and duration into PA assessment tools to improve reliability and comparability. Third, there were significant differences in the sample sizes across different seasons, although we used statistical methods such as adding weight variables to minimize the impact of these differences on the study results, caution is still needed when interpreting the season-related outcomes. Finally, as this study employs a cross-sectional design, it is unable to determine causality between school levels, urban–rural settings, regions, seasons, and PA or RST. Readers should bear this in mind when interpreting the results of this study.

## 5 Conclusion

From 2017 to 2019 in China, only 28.57% of children and adolescents adhered to the WHO’s PA guidelines, whereas 81.61% met the RST guidelines of China and Canada. Notably, females, older students, rural residents, and those in southern regions exhibited lower levels of PA compared to their male, younger, urban, and northern counterparts. Simultaneously, males, younger students, rural residents, and those in northern regions had longer periods of RST compared to females, older students, urban dwellers, and children and adolescents from southern regions. Furthermore, it was observed that, compared to the spring, children and adolescents engage in less PA and more RST during the autumn and winter. Consequently, targeted efforts are essential to enhance PA among females, older students, rural residents, and southerners, while simultaneously reducing RST among males, younger students, rural residents, and northerners. Additionally, it is crucial to develop PA facilities that are not affected by climatic conditions to increase PA and reduce RST among this demographic group.

## Data availability statement

The raw data supporting the conclusions of this article will be made available by the authors, without undue reservation.

## Ethics statement

The studies involving humans were approved by Human Experimentation Ethics Committee of East China Normal University. The studies were conducted in accordance with the local legislation and institutional requirements. Written informed consent for participation in this study was provided by the participants’ legal guardians/next of kin.

## Author contributions

MG: Data curation, Formal analysis, Funding acquisition, Methodology, Writing – original draft, Writing – review & editing. YZ: Data curation, Writing – review & editing. XW: Conceptualization, Writing – review & editing.
